# Differences in Vitamin A Levels and Their Association with the Atherogenic Index of Plasma and Subclinical Hypothyroidism in Adults: A Cross-Sectional Analysis in China

**DOI:** 10.3390/nu16162613

**Published:** 2024-08-08

**Authors:** Guangming Mao, Manman Chen, Lichun Huang, Zhe Mo, Danting Su, Simeng Gu, Fanjia Guo, Yuanyang Wang, Zhijian Chen, Ronghua Zhang, Xiaoming Lou, Xiaofeng Wang, Jie Hu, Fang Gu, Bin Dong

**Affiliations:** 1 Department of Environmental Health, Zhejiang Provincial Center for Disease Control and Prevention, 3399 Binsheng Road, Hangzhou 310051, China; gmmao@cdc.zj.cn (G.M.); zhmo@cdc.zj.cn (Z.M.); smgu@cdc.zj.cn (S.G.); fjguo@cdc.zj.cn (F.G.); yywang@cdc.zj.cn (Y.W.); zhjchen@cdc.zj.cn (Z.C.); xmlou@cdc.zj.cn (X.L.); xfwang@cdc.zj.cn (X.W.); 2 School of Population Medicine and Public Health, Chinese Academy of Medical Sciences and Peking Union Medical College, Beijing 100730, China; chenmm@pumc.edu.cn; 3 Institute of Nutrition and Food Safety, Zhejiang Provincial Center for Disease Control and Prevention, 3399 Binsheng Road, Hangzhou 310051, China; lchhuang@cdc.zj.cn (L.H.); dtsu@cdc.zj.cn (D.S.); rhzhang@cdc.zj.cn (R.Z.); 4 Menzies Health Institute Queensland, Griffith University, Brisbane, QLD 4111, Australia; jie.hu@griffith.edu.au; 5 Institute of Child and Adolescent Health, School of Public Health, Peking University Health Science Center, No. 38 Xueyuan Road, Haidian District, Beijing 100191, China

**Keywords:** vitamin A levels, atherogenic index of plasma, subclinical hypothyroidism, adults

## Abstract

Background: This study evaluates the association between vitamin A levels, AIP (the atherogenic index of plasma), and subclinical hypothyroidism. Methods: A cross-sectional analysis was conducted involving a representative sample of 3530 Chinese adults. Linear and logistic regression models were utilized to evaluate the associations between AIP and subclinical hypothyroidism, stratified by vitamin A levels. These analyses were further differentiated by sex and age groups to identify any demographic-specific associations. Results: In the vitamin A-sufficient group, an increase in AIP was associated with elevated total triiodothyronine (TT3) levels (*β* = 0.26, 95%CI: 0.09, 0.41, *p* = 0.003). Conversely, in the group with severe vitamin A deficiency, higher AIP levels were linked to increased free triiodothyronine (fT3) and TT3 levels and decreased free thyroxine (fT4) levels (*β* = 0.12, 0.03, and −0.29, respectively). Additionally, severe vitamin A deficiency increased the risk associated with AIP and subclinical hypothyroidism (OR = 1.66, 95%CI: 1.07, 2.58, *p* = 0.025). This risk was notably more pronounced in women and older adults, with odds ratios of 2.44 (95%CI: 1.55, 3.86, *p* < 0.001) and 2.14 (95%CI: 1.36, 3.38, *p* = 0.001), respectively. Conclusions: Vitamin A deficiency may increase the risk of the association between AIP and subclinical hypothyroidism, particularly among women and the elderly.

## 1. Introduction

Hypothyroidism, particularly in its subclinical form, presents a significant public health challenge, affecting various populations globally with notable endocrine and metabolic diseases [1,2]. Subclinical hypothyroidism, characterized by elevated thyroid-stimulating hormone (TSH) levels and normal thyroxine (T4) levels, often escapes early diagnosis due to its subtly manifesting symptoms [3,4]. Observational studies have demonstrated that subclinical hypothyroidism is associated with a 20–80% increase in vascular morbidity and mortality [5,6]. This association underscores the critical need for heightened awareness and early detection strategies to manage and mitigate the health risks associated with this frequently underdiagnosed condition.

The atherogenic index of plasma (AIP), calculated using the levels of triglycerides and high-density lipoprotein (HDL) cholesterol, has emerged as a critical predictive indicator for atherosclerosis and cardiovascular diseases [7,8]. AIP is essential in identifying individuals at heightened risk of cardiovascular disease, highlighting the intricate interplay between thyroid function, lipid metabolism, and cardiovascular health. By understanding these interactions, healthcare professionals can better predict and manage the health outcomes of their patients [9,10].

Recent studies have also begun to explore the role of micronutrients in thyroid function, with vitamin A emerging as a nutrient of interest due to its role in modulating thyroid hormone metabolism and immune function [11,12,13,14]. Vitamin A is known to regulate thyroid hormone metabolism and the pituitary–thyroid axis, potentially affecting thyroid hormone levels and overall thyroid function [15,16,17]. Moreover, thyroid hormones and vitamin A share a mutual association where thyroid hormones can influence the metabolism of vitamin A, suggesting a bidirectional relationship [18,19]. Despite the recognized importance of this interplay, the specific impact of vitamin A on subclinical hypothyroidism and related metabolic indices like AIP is not fully understood.

Considering the prevalent issues of vitamin A deficiency, AIP, and thyroid disorders globally, particularly in aging populations, this research could provide essential insights into nutritional interventions. To address the existing knowledge gaps, our study investigated the variations in vitamin A levels and their associations with AIP, thyroid hormones, and thyroid disease, through a cross-sectional analysis involving 3530 Chinese adults.

## 2. Materials and Methods

### 2.1. Study Design and Participants

In 2022, a cross-sectional survey was conducted using a multi-stage stratified random sampling method to select a representative sample of adults in Zhejiang Province. The survey’s sampling methodology was detailed in prior publications [20,21]. Zhejiang Province comprises eleven cities, encompassing a total of 90 counties (districts). Initially, 2 counties (or districts) were randomly chosen from each city, resulting in a total of 22 counties. Subsequently, 3 towns (or streets) were randomly selected from each of these counties, amounting to 66 towns or streets. Finally, one community was randomly selected from each town (or street).

Preliminary investigations led to the exclusion of subjects who, in the past six months, had been exposed to any of the following: iodinated contrast media for coronary angiography or endoscopic retrograde cholangiopancreatography, amiodarone drugs, or who had severe psychological disorders or dementia. A total of 3530 adults over 18 years old participated in this study. Participants under the age of 18 were excluded due to our focus on adult-onset subclinical hypothyroidism, which is less common among younger individuals. Additionally, ethical considerations and the unique physiological and nutritional needs of minors influenced this decision.

The Ethical Committee of the Zhejiang Provincial Chinese Centers for Disease Control and Prevention (CDC) granted ethical approval (approval code: 2022-018-01; approval date: 10 May 2022), and written informed consent was obtained from all participants.

### 2.2. Measurements and Classifications

The atherogenic index of plasma (AIP): AIP is calculated based on indicators derived from blood samples, specifically triglycerides (TGs) and high-density lipoprotein cholesterol (HDL-C). To ensure data reliability and comparability, stringent quality control measures were applied when collecting these clinical parameters. AIP is calculated by taking the logarithm base 10 of the ratio of TGs to HDL-C, both measured in molar concentration (mmol/L), expressed as [log10 (TG/HDL-C)] [22].

Thyroid hormones and diseases: For the investigation of thyroid hormones and diseases, approximately 5 mL of fasting venous blood was collected from one participant per household. After serum extraction through centrifugation, it was stored at −70 °C. Free triiodothyronine (fT3), free tetraiodothyronine (fT4), thyroid-stimulating hormone (TSH), and TSH receptor antibodies (TRAbs) were measured using radioimmunoassay techniques (Beijing Atom High Tech Co., Ltd., Beijing, China). Thyroglobulin antibodies (TgAbs) and thyroid peroxidase antibodies (TPOAbs) were assessed using the chemiluminescence immunoassay method via the Bayer ADVIA Centaur System (Bayer Healthcare, Leverkusen, Germany). Subclinical hypothyroidism was defined as TSH > 4.20 mIU/L, with fT4 being within the normal range. In this study, subclinical hypothyroidism is known as thyroid disease.

Vitamin A levels: Venous blood samples were collected and treated with separation gel to extract the supernatant. Vitamin A levels were quantified using liquid chromatography–tandem mass spectrometry. According to the World Health Organization (WHO) (https://www.who.int/data/nutrition/nlis/info/vitamin-a-deficiency, accessed on 25 July 2024), subclinical vitamin A deficiency in adults is defined as a plasma or serum retinol concentration below 0.70 μmol/L, while severe vitamin A deficiency is characterized by a concentration below 0.35 μmol/L.

### 2.3. Covariates

All participants underwent a comprehensive physical examination conducted by trained medical staff following a standardized protocol. Height and weight were measured by professional public health doctors. Body mass index (BMI) was determined by dividing the weight in kilograms (kg) by the square of the height in meters (m^2^). Smoking status was obtained through a questionnaire. Vitamin D levels were quantified using liquid chromatography–tandem mass spectrometry. Urine iodine levels were measured using the As^3+^-Ce^4+^ catalytic spectrophotometric method.

### 2.4. Statistical Analysis

Descriptive statistics were used to summarize the demographic characteristics of each participant. Continuous variables were described using the mean and standard deviation (SD), while categorical variables were reported as the frequency (proportion). To evaluate the differences in demographic characteristics between adults who reported sufficient vitamin A levels or deficiency, two-sample t-tests or Chi-square tests were conducted. The nonparametric Mann–Whitney test was applied to test the difference in thyroid hormones across vitamin A levels.

Linear regression models were applied to analyze the association between AIP and thyroid hormones, stratified by vitamin A levels. Binary logistic regression models were applied to analyze the association between AIP and thyroid diseases, stratified by vitamin A levels. Sensitivity analyses were performed to analyze the association between AIP with thyroid hormones and thyroid diseases across subclinical vitamin A deficiency and tertiles of vitamin A levels. Stratified analyses were also performed by sex and age groups. All analyses were adjusted for age, BMI, sex, smoking status, vitamin D levels, and urine iodine levels. Statistical analyses were performed with R (version 4.3.2, R Core Team, Vienna, Australia). Two-sided *p*-values < 0.05 were considered to indicate statistical significance.

## 3. Results

### 3.1. Participant Characteristics

The basic demographic characteristics of 3530 participants (1558 males vs. 1972 females) are presented in Table 1. There were 1620 (45.89%) participants in the age group below 50 years old and 1910 (54.11%) participants in the age group above 50 years old. There was a statistical difference in height, weight, and BMI between participants who were vitamin A-sufficient and those with severe vitamin A deficiency (*p* < 0.001). The mean value of AIP in the vitamin A-sufficient group was greater than in the severe vitamin A deficiency group (0.03 vs. −0.24), with a statistically significant difference between the two groups (*p* < 0.001). The proportions of subclinical hypothyroidism in the vitamin-sufficient and severe deficiency groups were 9.79%, and 11.62%, respectively, and the difference was not statistically significant (*p* = 0.554).

### 3.2. Differences in Vitamin A Levels and Their Association with AIP and Thyroid Hormones and Diseases

Table 2 displays the results of the linear and logistic models, which analyze the association between AIP and thyroid hormones and diseases, stratified by vitamin A levels. In the vitamin A-sufficient group, the study identified an association where an increase in AIP was associated with a rise in TT3 (*β* = 0.26, 95%CI: 0.09, 0.43, *p* = 0.003). In the severe vitamin A deficiency group, an increase in AIP can elevate the levels of fT3 (*β* = 0.12, 95%CI: 0.05, 0.18, *p* = 0.001) and TT3 (*β* = 0.03, 95%CI: 0.00, 0.07, *p* = 0.048), and decrease the fT4 levels (*β* = −0.29, 95%CI: −0.54, −0.03, *p* = 0.029). Furthermore, severe vitamin A deficiency may heighten the risk of the association between AIP and subclinical hypothyroidism (OR = 1.66, 95%CI: 1.07, 2.58, *p* = 0.025). Additionally, linear and logistic models were used to analyze the association between AIP and thyroid hormones and diseases across subclinical vitamin A deficiency and tertiles of vitamin A levels, as shown in Tables S1 and S2. Subclinical vitamin A deficiency may also heighten the risk of the association between AIP and subclinical hypothyroidism (OR = 1.69, 95%CI: 1.05, 2.73, *p* = 0.031). Additionally, in the lowest quartile (Q1) of vitamin A levels, it appears that lower vitamin A levels may increase the risk associated with AIP and subclinical hypothyroidism (OR = 1.91, 95%CI: 1.02, 3.60, *p* = 0.044).

### 3.3. Differences in Vitamin A Levels and Their Association with AIP and Thyroid Hormones and Diseases, Stratified by Sex and Age

**Figure 1:**
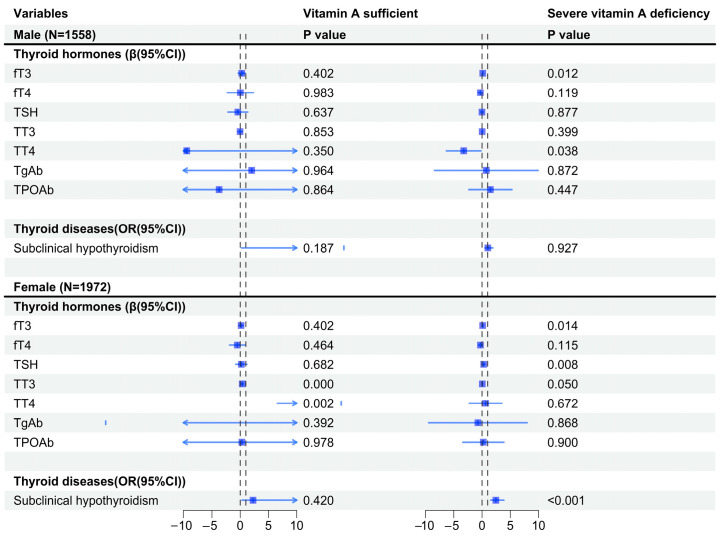
Vitamin A levels and their association with AIP and thyroid hormones and diseases, stratified by sex. Note: The model was adjusted for age, BMI, smoking, vitamin D levels, and urine iodine levels. fT3, free triiodothyronine; fT4, free tetraiodothyronine, TSH, thyroid stimulating hormone; TT3, total triiodothyronine; TT4, total thyroxine; TgAb, thyroglobulin antibody; TPOAb, thyroid peroxidase antibody. The arrow represents the range beyond the abscissa.

**Figure 2:**
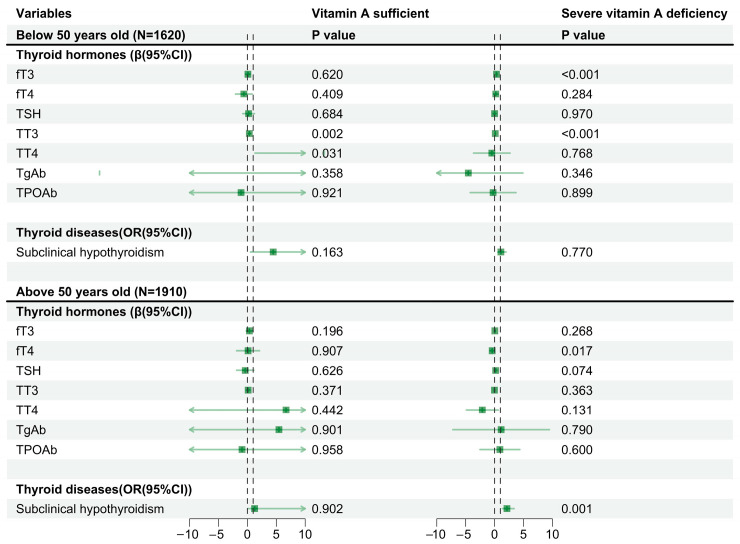
Vitamin A levels and their association with AIP and thyroid hormones and diseases, stratified by age. Note: The model was adjusted for sex, BMI, smoking, vitamin D levels, and urine iodine levels. fT3, free triiodothyronine; fT4, free tetraiodothyronine, TSH, thyroid stimulating hormone; TT3, total triiodothyronine; TT4, total thyroxine; TgAb, thyroglobulin antibody; TPOAb, thyroid peroxidase antibody. The arrow represents the range beyond the abscissa.

The results of linear and regression models regarding the association between AIP and thyroid hormones and diseases, stratified by different vitamin A levels, sex, and age, are shown in Figure 1 and Figure 2. In different sex groups, AIP increased the risk of subclinical hypothyroidism in women (OR = 2.44, 95%CI: 1.55, 3.86, *p* < 0.001) in the presence of severe vitamin A deficiency, but this phenomenon was not found in men (OR = 1.03, 95%CI: 0.55, 1.91, *p* = 0.927). Across different age groups, AIP increases the risk of subclinical hypothyroidism in older age groups (>50 years old) (OR = 2.14, 95%CI: 1.36, 3.38, *p* = 0.001) in the presence of severe vitamin A deficiency, but this phenomenon was not observed in the younger age group (<50 years old) (OR = 1.09, 95%CI: 0.60, 1.99, *p* = 0.770).

## 4. Discussion

Using data from a representative epidemiological survey of adults in Zhejiang Province, the present study provides compelling evidence of the modulatory role of vitamin A in the association between AIP and subclinical hypothyroidism. The findings indicate that adequate vitamin A levels may exert a protective effect on thyroid function, particularly in women and older adults. Sufficient vitamin A may influence metabolic processes associated with subclinical hypothyroidism, underscoring its potential significance in maintaining thyroid health.

Our analysis revealed that in individuals with sufficient vitamin A levels, an increase in AIP was positively associated with elevated TT3 levels. This suggests that adequate vitamin A may support the maintenance of normal thyroid function, even in the presence of higher AIP [23]. As TT3 is an active form of thyroid hormone, its elevation in the context of sufficient vitamin A could indicate a compensatory mechanism aimed at preserving metabolic homeostasis [24]. In contrast, in the group with severe vitamin A deficiency, higher AIP was associated with increased levels of both fT3 and TT3, along with decreased levels of fT4 [25]. This pattern indicates a disrupted thyroid hormone profile, where the body attempts to increase the active thyroid hormone (fT3 and TT3) possibly as a response to low fT4 levels [26]. This disruption could be due to the role of vitamin A in thyroid hormone metabolism, where deficiency may impair the conversion of T4 to T3, leading to an imbalance [27,28].

Vitamin A plays a multifaceted role in maintaining thyroid health through mechanisms affecting hormone synthesis, receptor activity, gland morphology, nutrient interactions, and immune function [29]. Research suggests that retinoic acid can influence TSH levels by modulating the hypothalamic–pituitary–thyroid axis, which controls the release and regulation of thyroid hormones essential for metabolism, growth, and development [30]. Furthermore, evidence shows that vitamin A is necessary for maintaining the normal structure and function of the thyroid gland. Deficiencies in vitamin A may lead to structural changes in the gland, potentially impairing its ability to produce thyroid hormones efficiently [31].

Moreover, our study identified a significant association between vitamin A deficiency and an increased risk of subclinical hypothyroidism in the presence of elevated AIP. This finding adds to the growing body of evidence suggesting a significant interplay between lipid profiles and vitamin status in thyroid health. Previous studies have indicated that vitamin A supplementation could offer therapeutic benefits for patients with hypothyroidism [32], which is consistent with our results. For instance, a study involving 80 female patients aged 20 to 45 years found that atherogenic indices were significantly higher in those with hypothyroidism (*p* < 0.001) [33].

The novelty of our study lies in examining the combined influence of vitamin A status and AIP levels in predicting subclinical hypothyroidism. This association was particularly pronounced among women and older adults, suggesting that these groups may be more vulnerable to the adverse effects of both vitamin A deficiency and subclinical hypothyroidism. Women, especially post-menopausal women, often experience hormonal shifts that could be exacerbated by inadequate dietary intake or absorption of essential nutrients, including vitamin A [34,35,36]. Similarly, aging is associated with changes in metabolic rates and nutrient processing, making older adults particularly susceptible to deficiencies and their consequent health impacts [37,38].

The mechanisms underlying these associations could involve several pathways [39,40]. Vitamin A is essential for the synthesis of thyroid hormones and the regulation of TSH secretion [41,42]. It also plays a role in the immune system, which can influence thyroid autoimmunity. Vitamin A deficiency might impair these processes, leading to altered thyroid function and increased susceptibility to subclinical hypothyroidism [43,44]. These findings have important clinical implications. The current study suggested that maintaining adequate vitamin A levels could serve as a modifiable factor to mitigate the risk of subclinical hypothyroidism, particularly in vulnerable populations such as women and the elderly. Public health strategies aimed at improving vitamin A status through dietary interventions or supplementation could potentially enhance thyroid health and reduce the incidence of thyroid-related disorders [45,46].

However, our study has some limitations. First, the cross-sectional design restricts our ability to establish causality. Longitudinal studies are necessary to validate the associations and investigate the causal pathways underlying these relationships. Secondly, while our sample is representative of the adult population in Zhejiang province, extending the generalizability of our findings to other populations necessitates further investigation. Different demographic and geographic characteristics can influence health outcomes, and factors such as ethnic diversity, socioeconomic status, and lifestyle variations across populations may affect the applicability of our results. Thirdly, despite adjusting for several potential confounders, the possibility of residual confounding remains. Genetic factors and environmental influences could also impact thyroid health and should be addressed in future studies. Additionally, this study is limited by the low variability in AIP and the prevalence rates of subclinical hypothyroidism and vitamin A deficiency within the sample. This restricts our ability to generalize findings and fully explore the associations between these parameters. Future research should consider including populations with higher or more diverse prevalence rates to enable a more comprehensive analysis of these associations.

## 5. Conclusions

In conclusion, this study highlights the association between vitamin A levels, AIP, and subclinical hypothyroidism. Our results indicate that vitamin A deficiency increases the risk of thyroid dysfunction in the context of elevated AIP, with significant implications for women and older adults. Future research should focus on the potential benefits of vitamin A supplementation in improving thyroid health and preventing subclinical hypothyroidism, thereby contributing to overall metabolic and cardiovascular health.

## Figures and Tables

**Table 1:** Basic demographic characteristics.

Variables	Vitamin A-Sufficient	Severe Vitamin A Deficiency	*p*-Value
Sex (N (%))			
Male	1515 (46.06)	43 (17.84)	<0.001
Female	1774 (53.94)	198 (82.16)	
Age group (N (%))			
Below 50 years old	1445 (43.93)	175 (72.61)	<0.001
Above 50 years old	1844 (56.07)	66 (27.39)	
Height (cm, mean ± SD)	162.96 ± 8.6	160.58 ± 6.61	<0.001
Weight (kg, mean ± SD)	63.11 ± 11.29	56.31 ± 8.75	<0.001
BMI (kg/m^2,^ mean ± SD)	23.71 ± 3.55	21.85 ± 3.26	<0.001
AIP	0.03 ± 0.33	−0.24 ± 0.24	<0.001
Thyroid hormones (median, IQR)			
fT3	4.82 (4.46, 5.24)	4.54 (4.25, 5.02)	<0.001
fT4	17.2 (15.8, 18.71)	16.58 (15.49, 17.93)	<0.001
TSH	2.03 (1.43, 2.93)	2.17 (1.45, 3.25)	0.150
TT3	1.71 (1.53, 1.9)	1.64 (1.46, 1.86)	0.009
TT4	109.2 (97.62, 121.3)	109.41 (98.72, 121)	0.160
TgAb	14.95 (12.86, 17.53)	15.63 (12.95, 19.77)	0.080
TPOAb	8.67 (6.63, 11.53)	8.25 (6.47, 11.09)	0.172
Subclinical hypothyroidism (N (%))			
No	2967 (90.21)	213 (88.38)	0.554
Yes	322 (9.79)	28 (11.62)	
Smoking status (N (%))			
No	2514 (76.44)	218 (90.46)	<0.001
Yes	775 (23.56)	23 (9.54)	
Vitamin D levels (N (%))			
Sufficient	741 (22.56)	23 (9.54)	<0.001
Deficiency	2543 (77.44)	218 (90.46)	
Urine iodine levels (N (%))			
Sufficient	1895 (67.20)	141 (70.50)	0.415
Deficiency	925 (32.80)	59 (29.50)	

Note: BMI, body mass index; AIP, atherogenic index of plasma; fT3, free triiodothyronine; fT4, free tetraiodothyronine, TSH, thyroid-stimulating hormone; TT3, total triiodothyronine; TT4, total thyroxine; TgAb, thyroglobulin antibody; TPOAb, thyroid peroxidase antibody.

**Table 2:** Differences in vitamin A levels and their association with AIP and thyroid hormones and diseases.

Variables	Vitamin A Sufficient			Severe Vitamin A Deficiency				
	Model 1		Model 2	Model 1			Model 2	
	***β* (95%CI)**	***p*-Value**	***β* (95%CI)**	***p*-Value**	***β* (95%CI)**	***p*-Value**	***β* (95%CI)**	***p*-Value**
Thyroid hormones								
fT3	0.18 (−0.11, 0.47)	0.217	0.18 (−0.13, 0.48)	0.257	0.21 (0.14, 0.27)	0.000	0.12 (0.05, 0.18)	0.001
fT4	−0.57 (−1.73, 0.58)	0.332	−0.38 (−1.56, 0.81)	0.536	−0.18 (−0.43, 0.07)	0.161	−0.29 (−0.54, −0.03)	0.029
TSH	0.13 (−0.67, 0.93)	0.753	0.06 (−0.80, 0.91)	0.894	0.10 (−0.05, 0.25)	0.175	0.14 (−0.01, 0.30)	0.074
TT3	0.27 (0.11, 0.43)	0.001	0.26 (0.09, 0.43)	0.003	0.07 (0.03, 0.10)	0.000	0.03 (0.00, 0.07)	0.048
TT4	10.09 (0.52,19.66)	0.040	11.29 (1.24, 21.34)	0.029	−1.59 (−3.59, 0.41)	0.120	−1.19 (−3.32, 0.94)	0.274
TgAb	−19.80 (−62.92, 23.32)	0.369	−16.80 (−63.45, 29.85)	0.481	−1.16 (−7.20, 4.88)	0.706	−0.03 (−6.43, 6.37)	0.993
TPOAb	0.96 (−16.53, 18.45)	0.914	−0.46 (−19.24, 18.33)	0.962	−0.34 (−2.85, 2.17)	0.790	0.82 (−1.84, 3.48)	0.544
	**OR (95%CI)**	***p*-Value**	**OR (95%CI)**	***p*-Value**	**OR (95%CI)**	***p*-Value**	**OR (95%CI)**	***p*-Value**
Thyroid diseases								
Subclinical hypothyroidism	3.15 (0.51, 19.32)	0.216	6.07 (0.81, 45.74)	0.080	4.13 (0.58, 29.25)	0.155	1.66 (1.07, 2.58)	0.025

Model 1 is crude; Model 2 is adjusted for age, BMI, sex, smoking, vitamin D levels, and urine iodine levels. fT3, free triiodothyronine; fT4, free tetraiodothyronine, TSH, thyroid-stimulating hormone; TT3, total triiodothyronine; TT4, total thyroxine; TgAb, thyroglobulin antibody; TPOAb, thyroid peroxidase antibody.

## Data Availability

The data are not publicly available due to privacy. The raw data supporting the conclusions of this article will be made available upon request.

## Supplementary Materials

The following supporting information can be downloaded at: https://www.mdpi.com/article/10.3390/nu16162613/s1, Table S1 Association between AIP and thyroid hormones and diseases, stratified by vitamin A levels; Table S2: Tertiles of the differences in vitamin A levels and their association with AIP and thyroid hormones and diseases.
