# Supplementary material for: Differences in Vitamin A Levels and Their Association with the Atherogenic Index of Plasma and Subclinical Hypothyroidism in Adults: A Cross-Sectional Analysis in China
Source: Nutrients. 2024 Aug 8;16(16):2613. doi: 10.3390/nu16162613 (PMC11357057; doi:10.3390/nu16162613)
Supplement: Supplementary file 1 [file nutrients-16-02613-s001.zip › nutrients-3117301-supplementary.pdf]

Supplemental materials

Table S1 Association between AIP and thyroid hormones and diseases, stratified by vitamin A levels

Variables	Vitamin A levels			
	Sufficient (>0.7)		Subclinical deficiency (<0.7)	
	β(95%CI)	*P* value	β(95%CI)	*P* value
**Thyroid hormones**				
fT3	0.15(0.00,0.31)	0.053	0.11(0.04,0.18)	0.003
fT4	-0.17(-0.82,0.48)	0.609	-0.39(-0.66,-0.11)	0.006
TSH	0.15(-0.24,0.53)	0.457	0.11(-0.06,0.29)	0.195
TT3	0.05(-0.03,0.13)	0.187	0.04(0.01,0.08)	0.015
TT4	-0.78(-5.89,4.32)	0.764	-0.45(-2.76,1.86)	0.702
TgAb	1.34(-13.06,15.75)	0.855	-2.12(-9.49,5.25)	0.573
TPOAb	1.28(-4.81,7.38)	0.680	0.12(-2.92,3.15)	0.940
	OR (95%CI)	*P* value	OR (95%CI)	*P* value
**Thyroid diseases**				
Subclinical hypothyroidism	1.24(0.43,3.59)	0.687	1.69(1.05,2.73)	0.031

Model was adjusted for age, BMI, sex, smoking, vitamin D levels, and urine iodine levels. fT3, free triiodothyronine; fT4, free tetraiodothyronine, TSH, thyroid-stimulating hor-mone; TT3, total triiodothyronine; TT4, total thyroxine; TgAb, thyroglobulin antibodies; TPOAb, thyroid peroxidase antibodies.

Table S2 Tertiles of vitamin A levels and the association between AIP and thyroid hormones and diseases

Variables	Tertiles of vitamin A levels					
	Q1		Q2		Q3	
	β(95%CI)	*P* value	β(95%CI)	*P* value	β(95%CI)	*P* value
**Thyroid hormones**						
fT3	0.15(0.03,0.26)	0.015	0.05(-0.05,0.15)	0.356	0.09(-0.04,0.23)	0.171
fT4	-0.18(-0.64,0.28)	0.449	-0.67(-1.06,-0.28)	0.001	-0.18(-0.71,0.35)	0.501
TSH	0.12(-0.15,0.40)	0.384	0.13(-0.12,0.38)	0.299	0.17(-0.16,0.51)	0.309
TT3	0.05(-0.01,0.11)	0.106	0.05(0.00,0.10)	0.040	0.04(-0.02,0.11)	0.204
TT4	-1.50(-5.20,2.19)	0.425	-0.38(-3.66,2.89)	0.819	4.08(-0.32,8.49)	0.069
TgAb	3.13(-7.41,13.66)	0.561	1.21(-8.81,11.23)	0.813	-10.01(-25.27,5.25)	0.199
TPOAb	-0.13(-4.86,4.60)	0.957	2.91(-1.10,6.92)	0.156	-4.62(-10.81,1.56)	0.143
	OR (95%CI)	*P* value	OR (95%CI)	*P* value	OR (95%CI)	*P* value
**Thyroid diseases**						
Subclinical hypothyroidism	1.97(1.05,3.68)	0.034	1.52(0.86,2.68)	0.154	1.98(0.89,4.41)	0.093

Model was adjusted for age, BMI, sex, smoking vitamin D levels, and urine iodine levels. fT3, free triiodothyronine; fT4, free tetraiodothyronine, TSH, thyroid-stimulating hor-mone; TT3, total triiodothyronine; TT4, total thyroxine; TgAb, thyroglobulin antibodies; TPOAb, thyroid peroxidase antibodies.
